# Worldwide Research Trends on Substance Use Disorder‐Related Inflammatory Imbalances: A Bibliometric Analysis

**DOI:** 10.1002/cns.70719

**Published:** 2025-12-20

**Authors:** Shirui Cao, Longtao Yang, Ruixin Wang, Jun Liu

**Affiliations:** ^1^ Department of Radiology The Second Xiangya Hospital of Central South University Changsha China; ^2^ Clinical Research Center for Medical Imaging in Hunan Province Changsha China; ^3^ Department of Radiology Quality Control Center in Hunan Province Changsha China

**Keywords:** addiction, gut microbiome, inflammation, N‐acetylcysteine, neuroinflammation, substance use disorders

## Abstract

**Background:**

Substance use disorder (SUD) is a complex, chronic, and relapsing encephalopathy associated with inflammatory processes. This study aims to conduct a bibliometric analysis to elucidate the current landscape, research hotspots, and evolving trends in the field of SUD‐related inflammation imbalance.

**Methods:**

Original research and review articles pertaining to “SUD‐inflammation” subject were systematically retrieved from the Web of Science Core Collection (WoSCC) database, encompassing publications from its inception to January 1, 2025. Co‐authorship, collaboration, co‐citation, and co‐occurrence analyses were performed using VOSviewer, and CiteSpace was utilized to identify strongest citation bursts and keyword trends.

**Results:**

A total of 2318 publications on “SUD‐inflammation” realm were included. A marked increase in publication output was observed starting in 2019. The United States (US) and China emerged as the most prolific countries. The journal, institution, and author, with the largest number of total publications (NP), were respectively JOURNAL OF ETHNOPHARMACOLOGY, UNIVERSITY OF CALIFORNIA SYSTEM, and Michael Maes. Core terms “microglial activation,” “oxidative stress,” and “TLR4 signaling” were among the most frequently studied topics. Additionally, the roles of the “gut‐immune‐brain” axis and N‐acetylcysteine in drug addiction have been receiving significant attention.

**Conclusion:**

This bibliometric study delineates the foundational knowledge structural and mapping of the “SUD‐inflammation” field over the past 25 years. The findings provide a comprehensive and data‐driven perspective on the evolving research landscape, offering valuable insights for future investigations and resource allocation.

## Graphical abstract

A bibliometric analysis of worldwide research trends on “substance use disorder‐related inflammation”. “Neuroinflammation” is identified as a key research hotspot. The findings provide a data‐driven overview of the evolving landscape in this field.

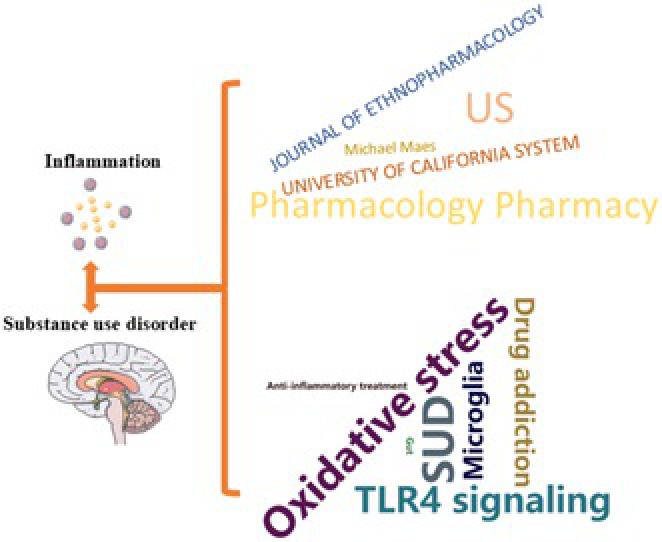

## 1 Introduction

Drug addiction, clinically known as substance use disorder (SUD), poses a major public health challenge with profound societal, economic, and medical implications. It is linked to heightened risk of mental illness, neurological disorders, infectious diseases, and criminal behavior, collectively undermining fundamental aspects of individuals' health and safety [1]. This chronic relapsing disorder is defined by cognitive, behavioral, and physiological disturbances, with core features including compulsive drug‐seeking despite awareness of the detrimental consequences of prolonged and excessive substance use [2, 3]. Recent epidemiological data from the United Nations Office on Drugs and Crime World Drug Report (2024) reports that approximately 64 million individuals worldwide are affected by SUD [4].

To mitigate the disease burden of SUD, researchers have persistently investigated the neurobiological mechanisms of drug addiction in pursuit of novel therapeutic interventions. Dopamine signaling in the mesolimbic reward circuit is well‐established in motivating and reinforcing substance addiction [5]. The mesolimbic reward circuit comprises dopaminergic neurons primarily located in ventral tegmental area (VTA) of the midbrain, which project to the nucleus accumbens (NAc), a key structure participated in processing reward‐related stimuli [6]. Recurrent drug abuse augments dopaminergic signaling transmission from VTA to NAc, thereby facilitating reward‐seeking behavior and elevating SUD risk [7]. However, owing to multifaceted nature of addiction mechanisms and genetic heterogeneity, fundamental molecular mechanisms underlying transition from initial substance reward to abuse remain elusive.

Conventional research in SUD concentrates on the direct effects of addictive substances on synaptic plasticity, dopamine system, and neural circuitry [8, 9]. Excitingly, neuroinflammation provided a novel perspective on the neurobiological mechanisms of drug addiction [10]. This Pathological neuroinflammation arises from resident innate immune cell activation in central nervous system (CNS), molecular signal release (e.g., cytokines, chemokines) [11], blood–brain barrier (BBB) disruption, and brain infiltration by activated immune cells [12]. Growing studies on drug addiction indicate that CNS immune signaling significantly modulates mesolimbic dopamine reward pathways induced by addictive drugs [13]. For example, Toll‐like receptor 4 (TLR4) signaling pathway is crucial for the rewarding and reinforcing effects of psychostimulants (e.g., cocaine and methamphetamine) [14, 15] and opioids (e.g., morphine) [16]. MyD88, an adaptor protein for TLR4, upregulates expression of pro‐inflammatory cytokines (e.g., Interleukin‐6 (IL‐6), IL‐1β), consequently stimulating dopamine release in the NAc [13, 17]. Although numerous studies have investigated inflammatory dysregulation in SUD from diverse perspectives, an updated overall research progress on this field is lacking. Through identification of research trends, gaps, and emerging areas within “SUD‐inflammation” domain, it is essential to align future research with clinical needs and public health priorities, ultimately improving SUD patient outcomes and reducing healthcare burdens.

Bibliometrics is a technique for employing mathematical and statistical principles to comprehensively summarize academic literature [18]. It enables quantitative analysis of a large volume of academic publications, thereby assessing academic influence of countries, journals, authors, and institutions in specific research fields [19]. Despite “SUD‐inflammation” interaction is gaining significant attention, no bibliometric analysis has been conducted in this field yet. During bibliometric analysis, scientific progress recognition, direction guiding, resource allocation, and collaboration facilitation can be proposed via identification of the most influential country, institution, author, journal, and publication [20]. Thus, this study aims to fill this gap by constructing a knowledge map of academic publications on SUD‐associated inflammatory imbalances.

## 2 Methods

### 2.1 Data Acquisition and Search Strategy

As a premier academic database, Web of Science (WoS), which encompasses over 12,000 high‐quality journals, provides extensive citation records and has been consistently selected as the target database across bibliometric studies [21, 22]. Therefore, on January 1, 2025, an exhaustive literature search was conducted on Web of Science Core Collection database (WoSCC), covering data from SCIE and SSCI. Synonyms and related terms were added to the keyword search to improve retrieval accuracy. The search terms were set as follows: TS = (“substance use disorder$” OR “heroin use disorder$” OR “opioid use disorder$” OR “methamphetamine use disorder$” OR “cannabis use disorder$” OR “marijuana use disorder$” OR “cocaine use disorder$” OR “ecstasy use disorder$”) AND TS = (“inflammation$” OR “inflammatory”). The “$” symbol in search terms is a wildcard that represents zero or one character, capturing singular or plural forms. The inclusion criteria of literature were: publications before January 1, 2025; English language conforming to the language type preferences of the majority of leading global journals; document types restricted to research articles and reviews. Publications were excluded using the following criteria: different article types (e.g., early access, correction, meeting abstract, editorial material, retraction, proceeding paper, letter, book chapters, or retracted publication).

### 2.2 Data Analysis

Bibliometrics, a study of scholarly publications, utilizes statistical data to describe publishing trends and emphasizes the relationships between published works [23]. Within medical domain, bibliometrics primarily serves to assess research overview of a certain field [24]. Bibliometric analyses are typically divided into two main categories: evaluative and relational [23]. Evaluative bibliometrics is used to acknowledge literature‐related information (e.g., journal impact factor (JIF), H‐index). Relational bibliometrics generates relationship networks between publications and their binding metadata (e.g., author, journal, institution).

In this work, VOSviewer and CiteSpace software were applied for bibliometric analyses. As for relational bibliometrics, in a form of network, VOSviewer visualizes term co‐occurrence, inter‐country/institution collaboration, inter‐authorship, and reference co‐citation through extraction of bibliometric information via association‐strength clustering algorithms, probabilistic and distance‐based visualization methods. In a network yielded by VOSviewer, the size of nodes reflects the occurring frequency of target subjects derived from literature messages, with larger circles denoting greater importance; the lines between nodes represent the links between them; the thickness of these lines signifies the strength of the links. As a Java application, CiteSpace reveals and visualizes dynamic patterns and emerging trends in scientific literature. It was used to picture evolution of the bibliometric network over time, specifically detecting the strongest citation bursts among highly cited keywords within the defined timeframe. We adhered to the primary procedural steps of CiteSpace, which include time slicing (1999–2025), thresholding (k = 5), modeling, pruning, merging, and mapping. Furthermore, a world map depicting publication distribution was created using “Bioinformatics” (https://www.bioinformatics.com.cn/).

### 2.3 Validity and Reliability

All citation data were exported from the ISI WoS database as .txt files and subsequently loaded into VOSviewer, CiteSpace, and Microsoft Excel for further analyses. Two reviewers independently assessed the titles and abstracts of all publications to screen for and exclude irrelevant studies. Discrepancies between reviewers were resolved by consulting a third reviewer.

## 3 Results

### 3.1 Characterization Analysis

**FIGURE 1:**
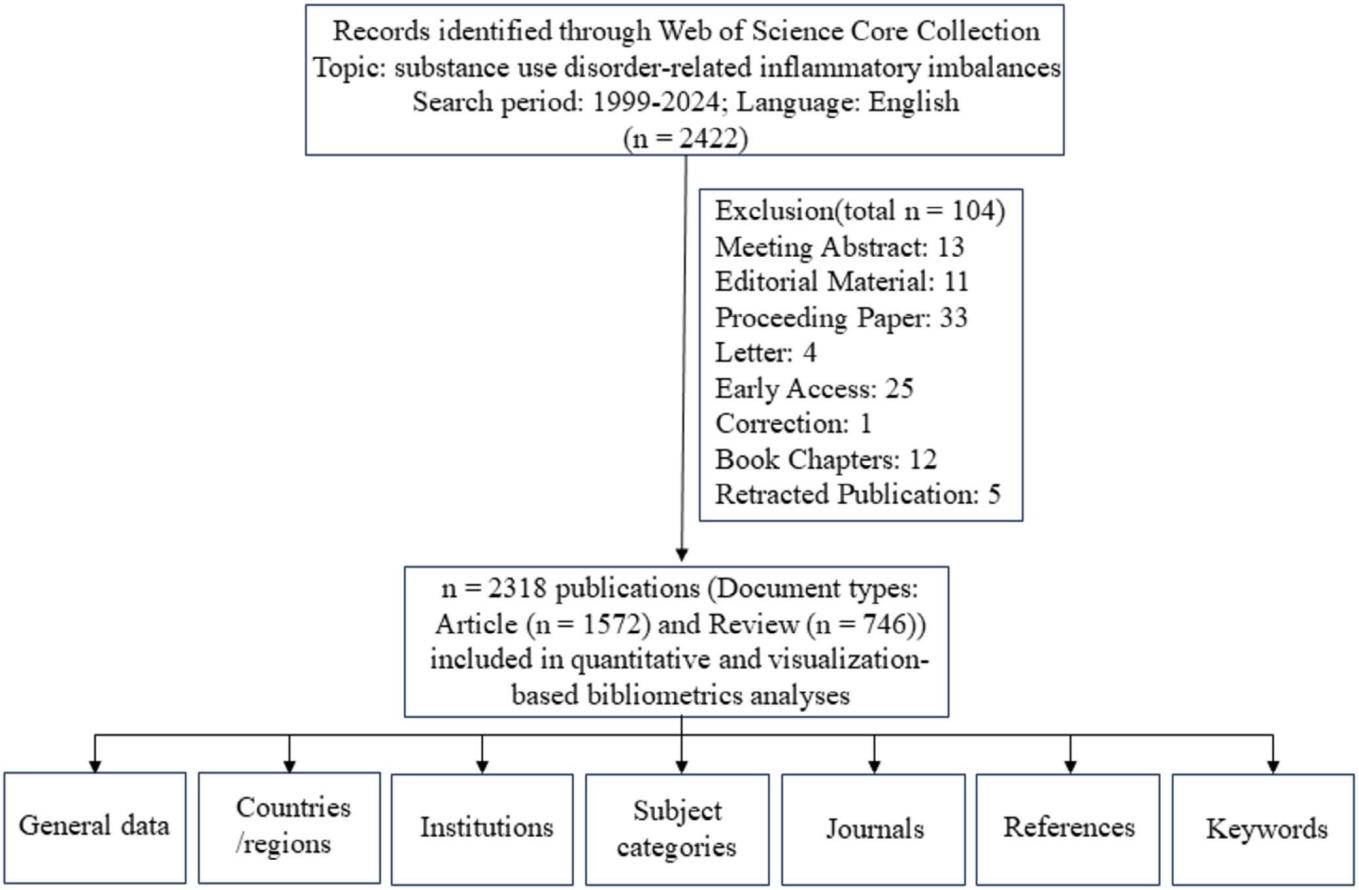
The flow chart of the study.

A total of 2422 publications in the WoSCC database before January 1, 2025 were included initially. Then, articles that did not meet the predefined inclusion criteria were excluded, resulting in a final 2318 publications, involving 1572 research articles and 746 reviews. The articles not classified as articles or reviews were excluded, comprising the following categories: Meeting Abstract, Editorial Material, Proceeding Paper, Letter, Early Access, Correction, Book Chapters, and Retracted Publication. For all included publications, we performed bibliometric analyses of the following dimensions: general data, country/region, institution, subject category, journal, reference, and keyword. The flowchart is shown in Figure 1.

### 3.2 Trajectories of the Numbers of Publications and Citations

**FIGURE 2:**
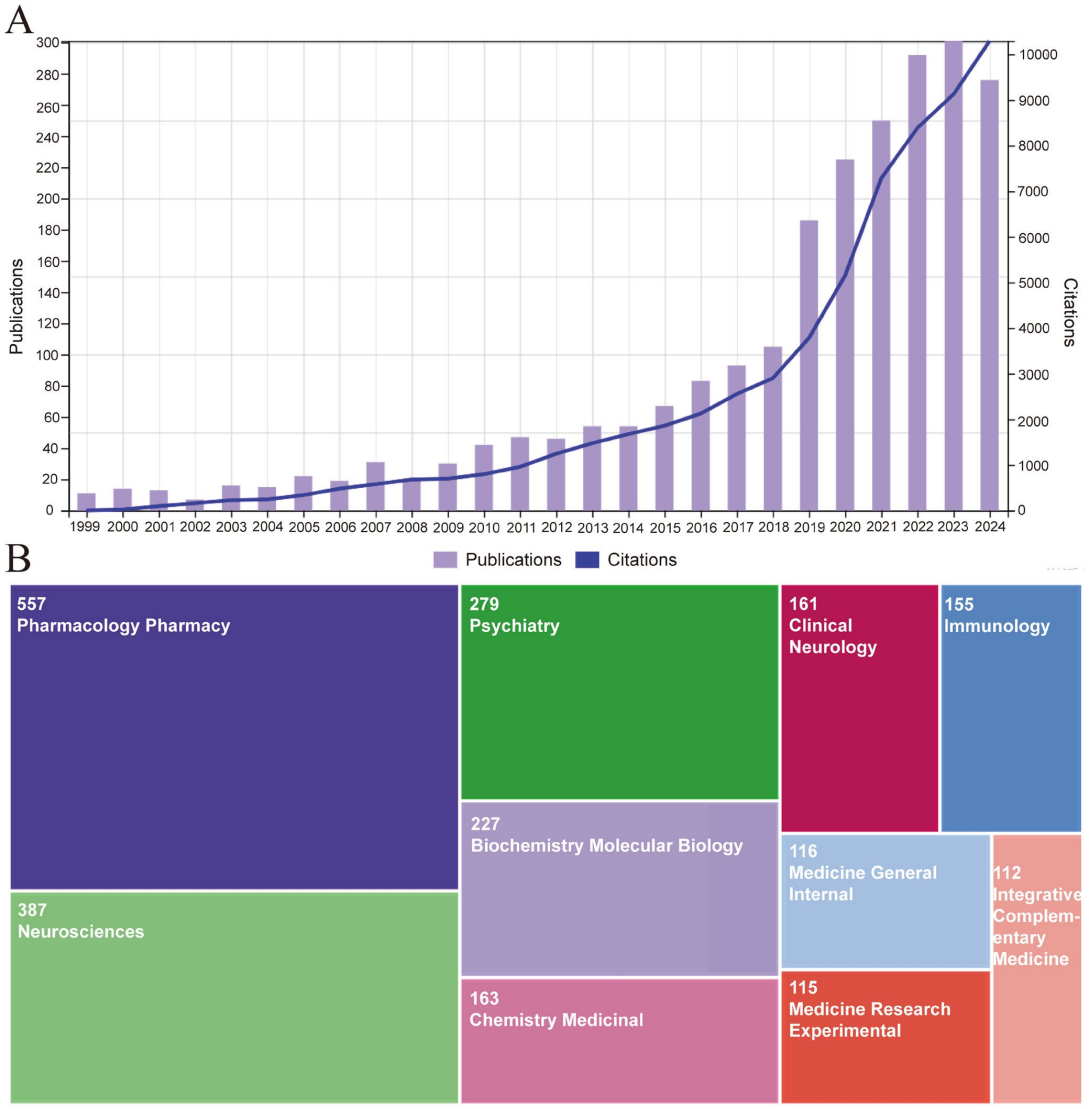
Trend of the numbers of publications and citations from 1999 to 2024 (A) as well as the distribution of publications across Web of Science categories (B).

Figure 2A illustrates the number and trend of annual publications and citations on SUD‐related inflammatory imbalances. A gradual increase in publications was observed from 1999 to 2014, while most annual outputs remained below 50 throughout this phase. During this period, this field was in a stage of conceptual exploratory groundwork. Starting in 2015, the annual number of total publications (NP) grew steadily at over 10%, surpassing 100 by 2018. A significant surge occurred in 2019, with over 180 literature and a growth rate exceeding 60%. Subsequent growth remained steady through 2023. A slight decline was observed in 2024, but its publications remained above 260. Number of total citations (NC) rose gradually from 1999 to 2014, remaining below 2000 annually. A marked increase commenced in 2015, coinciding with growing attention within this field. Since 2018, the NC growth rate accelerated dramatically, reaching more than 10,000 by 2023.

### 3.3 Publication Distribution Across WoS Categories

Figure 2B exhibits the top 10 scientific fields belonging to this research direction. Pharmacology Pharmacy owned the highest NP (*n* = 557, 24.0%), followed by Neurosciences (*n* = 387, 16.7%), Psychiatry (*n* = 279, 12.0%), and Biochemistry Molecular Biology (*n* = 227, 9.8%). Other notable fields included Chemistry Medicinal, Clinical Neurology, Immunology, Medicine General Internal, Medicine Research Experimental, and Integrative Complementary Medicine.

### 3.4 Publication Distribution Across Countries

**FIGURE 3:**
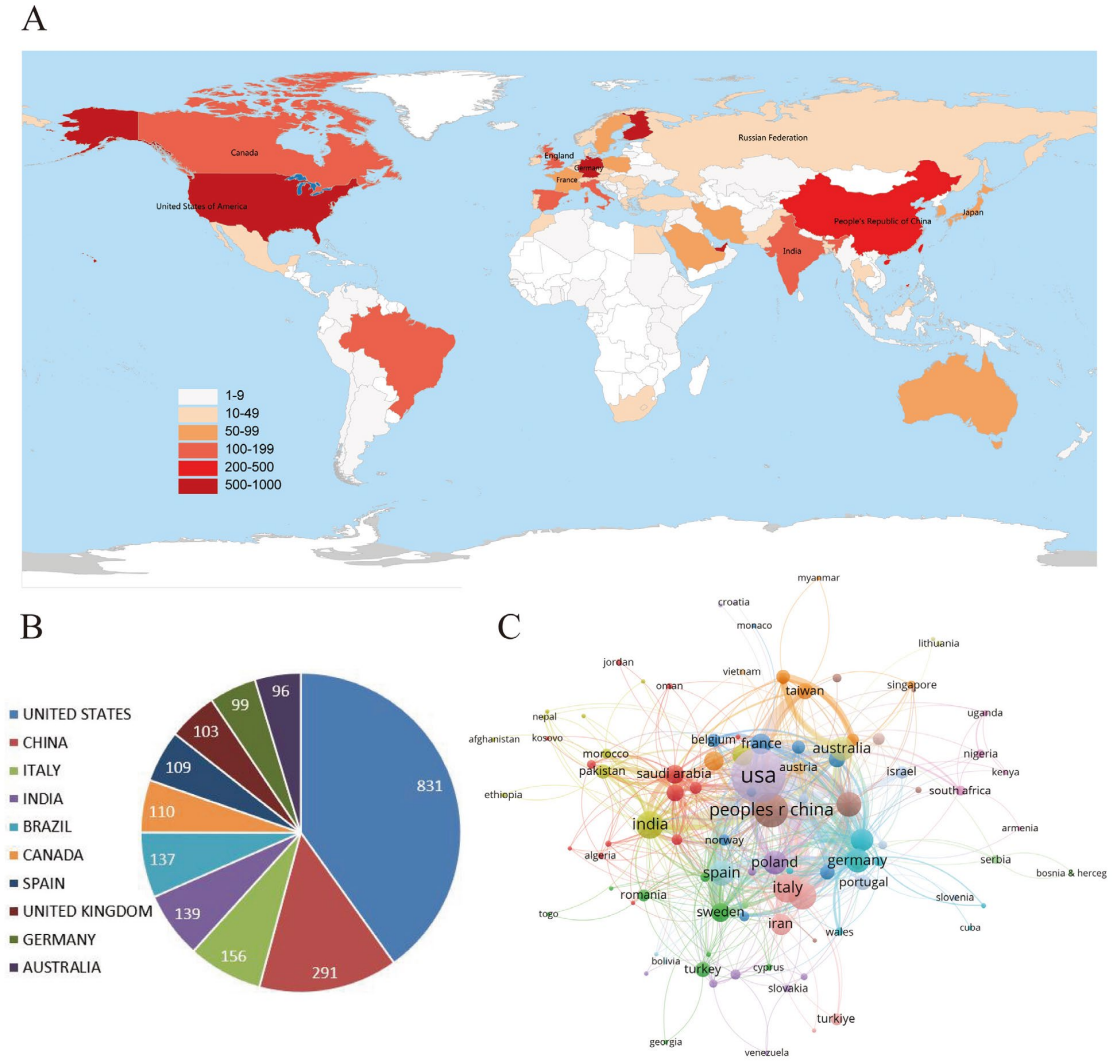
Global research on “substance use disorder‐related inflammation.” (A) Distribution of the total number of publications by country. (B) The top 10 most productive countries according to the number of publications. (C) Visualization of collaborative relationships of countries.

Figure 3A,B display the NP from different countries. The United States (US) leads with the highest NP (*n* = 831), followed by China (*n* = 291) and Italy (*n* = 156). Figure 3C presents the collaborative network among countries. The US dominates this field, boasting the largest publications and the most connections with other countries. European Countries (e.g., Italy, Sweden, Germany), China, Saudi Arabia, and India also positively cooperated with other countries. Table S1.1 shows NP per year of the top 10 countries over the past 10 years.

### 3.5 Publication Distribution Across Journals

Table 1 lists the top 10 journals ranked by NP. The most popular journal was *JOURNAL OF ETHNOPHARMACOLOGY* (*n* = 57), followed by *INTERNATIONAL JOURNAL OF MOLECULAR SCIENCES* (*n* = 42). Only 12.86% of papers were published in the top 10 journals. Citations per article (CA) is a quantitative indicator of a publication's influence; a higher CA signifies greater impact. Despite publishing fewer papers than *JOURNAL OF ETHNOPHARMACOLOGY* , journals including *MOLECULES, BRAIN* *BEHAVIOR AND* *IMMUNITY*, and *PLOS ONE* played a significant role in defining the research landscape of “SUD‐related inflammatory imbalances”. Particularly, *MOLECULES* emerged as the most influential journal in this area with a CA of 43.4. Table S1.2 shows NP per year of the top 10 journals over the past 10 years.

**TABLE 1:** Quantitative measurement of journal publishing research on SUD‐related inflammation.

Journal	NP	NC	Country	JIF (2023)	CA	H‐index
JOURNAL OF ETHNOPHARMACOLOGY	57	1713	Ireland	4.8	30.19	22
INTERNATIONAL JOURNAL OF MOLECULAR SCIENCES	42	718	Switzerland	4.9	17.17	13
MOLECULES	35	1516	Switzerland	4.2	43.4	20
FRONTIERS IN PHARMACOLOGY	29	515	Switzerland	4.4	17.79	13
PLOS ONE	25	1054	USA	2.9	42.16	16
FRONTIERS IN IMMUNOLOGY	23	513	Switzerland	5.7	22.3	11
NUTRIENTS	23	359	Switzerland	4.8	15.61	11
BRAIN BEHAVIOR AND IMMUNITY	22	906	USA	8.8	41.41	13
FRONTIERS IN PSYCHIATRY	22	418	USA	3.2	19.05	10
DRUG AND ALCOHOL DEPENDENCE	20	407	Switzerland	3.9	20.8	12

Abbreviations: CA, citations per article; NC, number of total citations; NP, number of total publications; SUD, substance use disorder.

### 3.6 Publication Distribution Across Institutions

Table 2 shows top 10 most productive institutions, All from the US. UNIVERSITY OF CALIFORNIA SYSTEM published the most papers (*n* = 88), but UNIVERSITY OF NORTH CAROLINA had the highest CA score (44.8). Co‐authorship analysis of institutions was visualized in S‐Figure S1.A. The US hosts the largest number of institutions, which collectively produce the highest NP and engage in broadly domestic collaboration. Notably, institutional collaboration primarily occurs within the same country, rather than across international borders. Table S1.3 shows NP per year of the top 10 institutions over the past 10 years.

**TABLE 2:** Quantitative measurement of top 10 institutions on SUD‐related inflammation.

Affiliations	NP	NC	Country	CA	H‐index
UNIVERSITY OF CALIFORNIA SYSTEM	88	2726	USA	31.15	25
HARVARD UNIVERSITY	59	2495	USA	42.32	22
PENNSYLVANIA COMMONWEALTH SYSTEM OF HIGHER EDUCATION PCSHE	59	1767	USA	30.37	21
NATIONAL INSTITUTES OF HEALTH NIH USA	48	2094	USA	43.85	21
US DEPARTMENT OF VETERANS AFFAIRS	48	1565	USA	32.73	23
VETERANS HEALTH ADMINISTRATION VHA	48	1565	USA	32.73	23
HARVARD MEDICAL SCHOOL	44	1739	USA	39.57	19
UNIVERSITY OF TEXAS SYSTEM	41	856	USA	20.98	17
UNIVERSITY OF NORTH CAROLINA	40	1790	USA	44.8	17
UNIVERSITY SYSTEM OF OHIO	39	1461	USA	37.62	13

Abbreviations: CA, citations per article; NC, number of total citations; NP, number of total publications; SUD, substance use disorder.

### 3.7 Top 10 Contributing Authors

As shown in Table 3, the most prolific researchers included one from Thailand, five from Spain, and four from the US. The research group led by Maes, Michael (Thailand) produced the highest NP (*n* = 11). Nevertheless, the most influential scholar was Theoharides, Theoharis C. From Tufts University School of Medicine (CA = 51.4, H‐Index = 9). S‐Figure S1B illustrates collaboration networks among researchers, revealing that the majority of collaborating authors are affiliated with Spanish institutions. Within the distinct collaborative clusters, authors in the red community exhibit the strongest collaborative ties and maintain connections with authors in the green and blue clusters. Table S1.4 shows NP per year of the top 10 authors over the past 10 years.

**TABLE 3:** Quantitative measurement of top 10 authors on SUD‐related inflammation.

Author	NP	NC	Country	Affiliations	CA	H‐index
Maes, Michael	11	184	Thailand	Chulalongkorn University	17.09	8
Serrano, Antonia	10	195	Spain	Hospital Regional Universitario de Málaga	21.6	7
Knapp, Pamela E.	10	231	USA	Virginia Commonwealth University	24	7
Theoharides, Theoharis C.	10	502	USA	Tufts University School of Medicine	51.4	9
Rodriguez‐Fonseca, Rolando Alberto	10	190	Spain	Hospital Regional Universitario de Málaga	21.4	7
Hong, Jau‐shyong	9	256	USA	NIH/NIEHS, Research Triangle Park	28.89	7
Hauser, Kurt	9	210	USA	Virginia Commonwealth University	24.33	7
Pavón Morón, Francisco Javier	8	201	Spain	Hospital Regional Universitario de Málaga	26.88	7
GÓMEZ, PEDRO FERNANDO ARAOS	8	201	Spain	Hospital Regional Universitario de Málaga	26.88	7
Garcia‐Marchena, Nuria	8	163	Spain	Hospital Universitari Germans Trias i Pujol	22.13	6

Abbreviations: CA, citations per article; NC, number of total citations; NP, number of total publications; SUD, substance use disorder.

### 3.8 Keyword Co‐Occurrence, Citation Bursts, and Timeline Distribution

**FIGURE 4:**
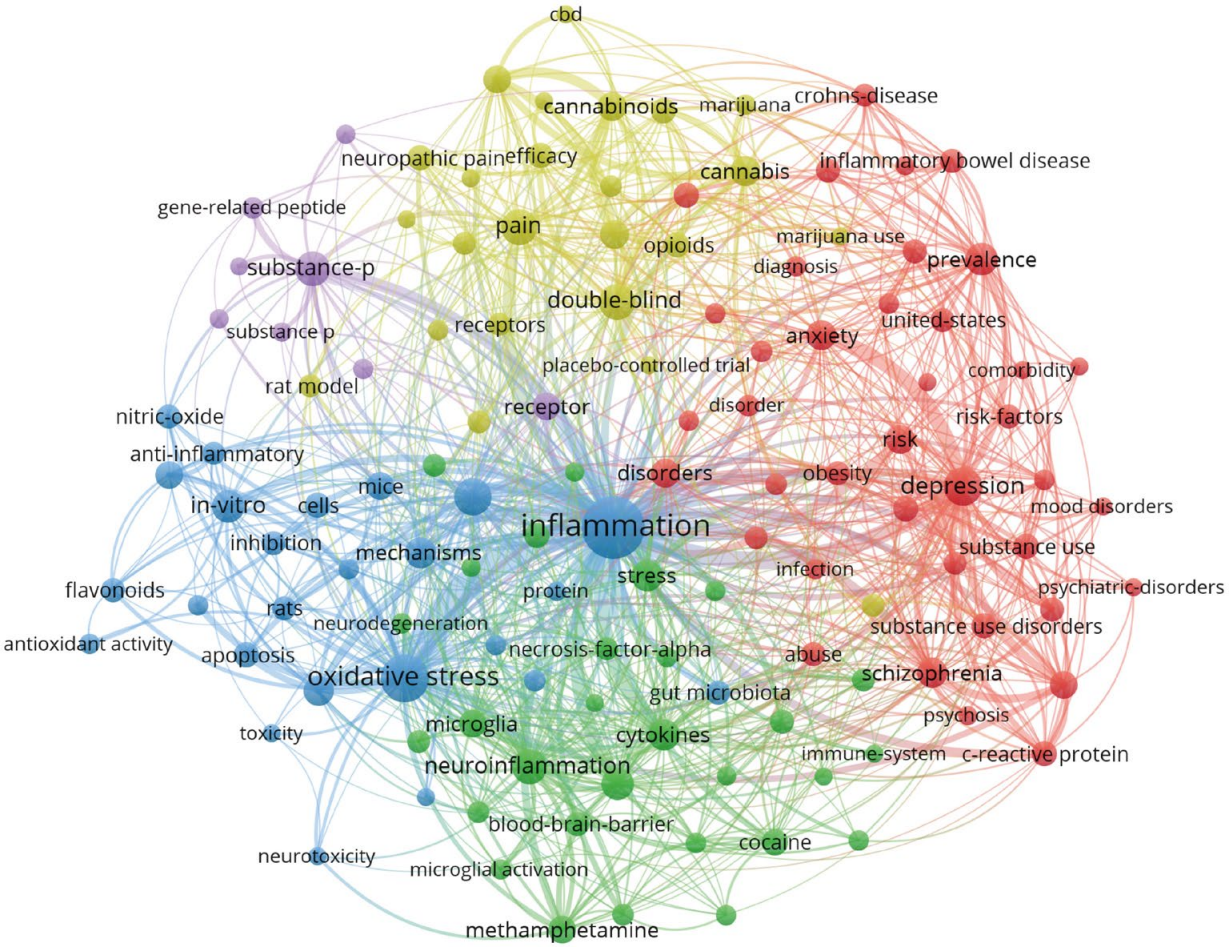
The keyword co‐occurrence network.

Figure 4 shows the network of keyword co‐occurrence. At a threshold of 25, we selected a total of 146 top keywords by minimum overlap set method. In Figure 4, these terms were categorized into five distinct thematic clusters, each uniquely color‐coded. Terms sharing the strongest co‐occurrence were assigned to the same cluster. The first cluster (blue) predominantly reflected pathophysiological processes involving inflammation and oxidative stress, accompanied by other prominent terms (e.g., antioxidant activity, apoptosis, neurodegeneration, neurotoxicity). The second cluster (red) was associated with psychiatric disorders (e.g., depression, schizophrenia). The third cluster (yellow) focused on substance‐related terms (e.g., marijuana, opioids). The fourth cluster (green) centered on neuroinflammatory mechanisms, incorporating terms (e.g., cytokines, necrosis‐factor‐alpha, blood–brain‐barrier, microglia activation). The fifth cluster (purple) was related to substance‐p. S‐Figure S2 shows co‐occurrence networks centered on “substance use disorders”, “opioid,” “methamphetamine,” “cannabis,” and “cocaine.”

**FIGURE 5:**
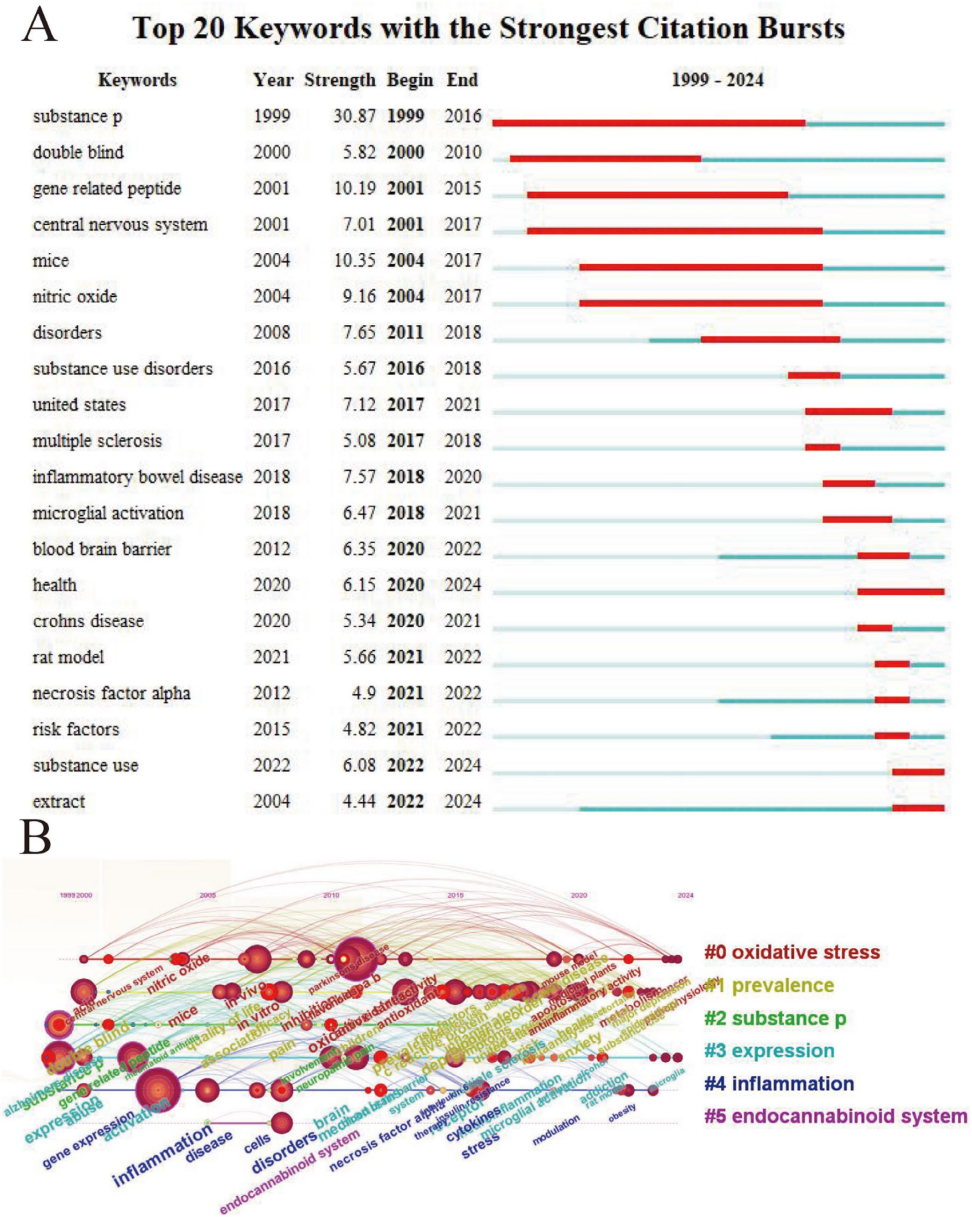
Top 20 keywords with the strongest citation bursts (A) and the timeline distribution of the top clusters of keywords (B).

Figure 5A shows top 20 keywords with the strongest citation bursts. The keywords “substance‐p” (1999–2016), “double blind” (2000–2010), “gene related peptide” (2001–2015), and “central nervous system” (2001–2017) received sustained attention during the initial phase of the study period. Keywords including “microglial activation” (2018–2021), “blood brain barrier” (2020–2022), and “necrosis factor alpha” (2021–2022) have been the most prevalent in recent years. The timeline distribution and clustering of these keywords are drawn in Figure 5B, which implies that oxidative stress represents a long‐standing research focus, while microglial activation has emerged as a prominent area of recent investigation.

### 3.9 Top 10 Most Cited Publications

Table 4 lists the top 10 most cited papers addressing inflammatory imbalances in SUD. The most cited work, by berk et al., titled “The promise of N‐acetylcysteine in neuropsychiatry” ( *TRENDS IN PHARMACOLOGICAL SCIENCES* ) has received 329 citations, averaging 27.42 citations per year [25]. Table S1.5 shows NP per year of the top 10 most cited publications over the past 10 years, reporting peak citation years primarily in 2019 and 2021. Figure S1C displays the co‐citation network among publications.

**TABLE 4:** Top 10 most cited publications.

Article titles	Author (year)	Source title	Citations/year	Citations
The promise of N‐acetylcysteine in neuropsychiatry	Berk et al. (2013)	TRENDS IN PHARMACOLOGICAL SCIENCES	27.42	329
Neuroinflammation in addiction: A review of neuroimaging studies and potential immunotherapies	Kohno et al. (2019)	PHARMACOLOGY BIOCHEMISTRY AND BEHAVIOR	17.5	105
Drugs of Abuse, Dopamine, and HIV‐Associated Neurocognitive Disorders/HIV‐Associated Dementia	Purohit et al. (2011)	MOLECULAR NEUROBIOLOGY	7.21	101
N‐acetylcysteine in the treatment of psychiatric disorders: current status and future prospects	Minarini et al. (2017)	EXPERT OPINION ON DRUG METABOLISM & TOXICOLOGY	11.25	90
Plasma profile of pro‐inflammatory cytokines and chemokines in cocaine users under outpatient treatment: influence of cocaine symptom severity and psychiatric co‐morbidity	Araos et al. (2015)	ADDICTION BIOLOGY	8.2	82
Glial and neuroinflammatory targets for treating substance use disorders	Bachtell et al. (2017)	DRUG AND ALCOHOL DEPENDENCE	9.13	73
Innate immune signaling in the ventral tegmental area contributes to drug‐primed reinstatement of cocaine seeking	Brown et al. (2018)	BRAIN BEHAVIOR AND IMMUNITY	9.86	69
N‐Acetylcysteine for the Treatment of Psychiatric Disorders: A Review of Current Evidence	Ooi et al. (2018)	BIOMED RESEARCH INTERNATIONAL	8.71	61
Neuroimmune mechanisms of psychostimulant and opioid use disorders	Hofford et al. (2019)	EUROPEAN JOURNAL OF NEUROSCIENCE	8.83	53
HIV‐1 gp120 and Drugs of Abuse: Interactions in the Central Nervous System	Silverstein et al. (2012)	CURRENT HIV RESEARCH	4	52

## 4 Discussion

This study employs bibliometrics to examine literature on inflammatory imbalances associated with SUD from 1999 to 2024. The field's development comprises three distinct phases: “slow development,” “steady growth,” and “rapid expansion” with a significant surge in annual publication output beginning in 2019.

### 4.1 Distribution of Countries, Institutions, and Authors

The top 10 countries account for 89.3% of total publications, with the US leading at 35.8%. In 2023, the US recorded the highest per capita healthcare expenditure ($13,432) among Organization for Economic Co‐operation and Development nations [26]. This substantial government investment in healthcare is a key contributor to the country's leadership position. In contrast, no Chinese or Italian institution ranks among the top 10, despite their second and third positions in publication volume, respectively. Institutional collaborations are more frequent within national borders, underscoring the imperative to bolster international interaction. Among the top 10 most productive authors, five are from Spain. The collaboration network demonstrates strong ties among Spanish authors, suggesting that inter‐author collaboration is a crucial pathway for enhancing research output and academic influence.

Geographically, the majority of publishers of the top 10 journals are headquartered in Switzerland and the US. To evaluate journal quality and impact, bibliometric indicators including JIF and H‐index were used. The JIF quantifies journal influence by calculating the average citation count per publication [27]. The H‐index, defined as h papers being cited at least h times each, measures researcher productivity and citation impact, balancing scholarly output with influence [28]. However, annual JIF is a short‐term metric for estimating journal quality, and other indicators (e.g., CA, H‐index) provide complementary insights. For instance, *PLOS ONE* ranked fifth in publication output, has a relatively low JIF (2.9 in 2023) yet demonstrates high academic value through its substantial CA and H‐index (CA = 42.16, H‐index = 16), reflecting its influential contributions to SUDs and inflammation research. Similarly, *MOLECULES* (CA = 43.3, H‐index = 20) and *BRAIN BEHAVIOR AND IMMUNITY* (CA = 41.41, H‐index = 13) exhibit high publication quality, underscoring their scholarly impact.

### 4.2 Hotspot Analysis—Based on Citations and Keywords

Citation and keyword analyses are instrumental in recognizing research hotspots and trends. Our findings highlight the evolutionary trajectory of research foci within the SUD‐inflammation domain. The terms such as “microglial activation” and “necrosis factor alpha,” which are linked to neuroinflammation, exhibit recent citation bursts. Similarly, keyword co‐occurrence analysis identifies frequent co‐occurrence of neuroinflammation‐related terms (e.g., oxidative stress) with addictive substances.

Neuroimmune systems (especially TLR4 signaling, microglia, oxidative stress) play a pivotal role in drug addiction progression. Stimulation of TLR4 signaling by drugs (e.g., psychostimulants, opioids, alcohol) strengthens proinflammatory reactions mainly through microglial activation, underlying the biological mechanisms of reward behaviors [29]. For instance, it reported that activation of VTA TLR4, primarily expressed by microglia, drove a moderate increase in cocaine seeking [30]. Long‐term alterations in TLR4‐driven microglial function of NAc, resulting from adolescent morphine exposure, increase the risk of morphine‐induced reinstatement in adulthood [31]. Then, microglial activation promotes the release of reactive oxygen species, chemokines, and pro‐inflammatory cytokines, which up‐modulate the functionality and expression of α‐amino‐3‐hydroxy‐5‐methyl‐4‐isoxazole‐propionic acid and N‐methyl‐D‐aspartic acid receptors and down‐modulate the expression of γ‐aminobutyric acid receptors and glutamate transporters [7]. These inflammatory mediators also boost neurotransmitter release and synaptic transmission, thereby increasing neuronal excitability at multiple nodes within neural circuitry involved in substance abuse [32]. Moreover, CNS oxidative stress initiated by substances (e.g., cocaine, opioids, cannabis, methamphetamine) can cause neuroinflammation [33]. Drug‐induced redox imbalance upregulated dopamine release [33]. Excessive reactive oxygen species (ROS) produced owing to the glutathione elimination in mitochondria promoted neuronal dopamine oxidation, which further advanced ROS generation and resulted in neurotoxicity [34].

Substance‐specific inflammatory pathways might be largely attributed to distinct effect properties. For instance, heroin acts on opioid receptors, while psychostimulants (e.g., amphetamines, cocaine) promote dopamine release via reversal or inhibition of dopamine transporter [35]. Alcohol enhances γ‐aminobutyric acid (GABA) inhibitory effect and dopamine release [36]. Opioid‐induced peripheral hypoxia triggers fragile‐like Treg phenotype [37]. Treg‐derived Interferon‐γ regulated withdrawal symptoms by reshaping synaptic morphology in NAc neurons [37]. Independent of dopamine release, cocaine could be directly involved in pattern recognition receptor signaling and cause an inflammatory response [17]. In alcohol‐dependent mice, amygdala IL‐10 overexpression modulated GABA transmission and abolished escalation of alcohol intake [38]. However, there may be commonalities in the inflammatory cascades that impact behavioral responses to distinct substance abuses. For example, an increase in brain tumor necrosis factor‐α can enhance withdrawal symptoms to psychostimulants [39] and opioids [40]. However, owing to confronted challenges (e.g., drug source, experimental operability), the in‐depth molecular biological mechanisms of “SUD‐inflammation” are still not explicit, and comprehensive investigations will be vital for latent clinical translation.

The “gut‐inflammation‐brain” axis is another area that has been receiving attention in SUD [41]. Communication between the gut and brain is bidirectional: vagal and spinal visceral afferent pathways; sympathetic and parasympathetic inputs [42]. The crosstalk of “gut‐SUD” signaling can be mediated by enteric inflammatory mechanisms (e.g., TLRs, macrophages) [43, 44]. Specifically, opioids altered gut microbial dysbiosis and impaired intestinal barrier function, leading to sustained inflammation of host [45]. Gut microbiome abundance correlated with opioid use and dependence via changing brain neuronal ensemble activation in a regional and state‐specific manner [46]. Morphine‐stimulated TLR signaling in intestinal epithelial cells caused epithelial barrier dysfunction via myosin light chain kinase pathways [47]. Besides, gut microbiome depletion promoted sensitivity to the sensitizing and rewarding effects of cocaine at low doses [48] and expansion of pro‐inflammatory factors in prefrontal cortex and hippocampus of methamphetamine‐exposed mice [49], as well as affected gene expression in the NAc, involving elevated levels of D1 and D2 dopamine receptors and brain‐derived neurotrophic factor [50]. Interestingly, neuroimaging research offers another approach to integrate “gut‐brain” axis in clinical studies of SUD [51]. While more work deserves to be done, evidence for gut‐immune‐brain connection in drug addiction is mounting.

Scientific landscape (e.g., most cited publication) captured by bibliometrics provides powerful quantitative lens to understand the research advancement, which will aid in guiding future direction of clinical trial progress and drug application. As a glutamatergic agent, N‐acetylcysteine (molecular formula: C_5_H_9_NO_3_S) that is taken as N‐acetyl prodrug acting on glutamatergic system has potential applications in the treatment of psychiatric disorders like SUD [3]. Preclinical studies have investigated the efficacy of N‐acetylcysteine in restoring normal glutamate signaling and reversing addiction pathology [52], which may also involve regulation of oxidative stress [53], glutamate levels [54], and dopamine system [55]. Although N‐acetylcysteine, as an adjunctive treatment drug holding potential for managing SUD, exhibits favorable safety and tolerability profiles [56], its clinical efficacy has not yet been fully established. Numerous studies have explored other potential therapeutic strategies for drug addiction from a neuroinflammatory perspective. For example, Ibudilast suppressed methamphetamine intake and motivation by inhibiting TLR4 signaling pathway as well as release of IL‐6 and p‐nuclear factor kappa‐B in the prefrontal cortex [57]. The lipid‐derived messenger oleoylethanolamide exerted a protective effect on stress‐mediated enhanced cocaine sensitivity through down‐regulation of TLR4 pathway [58]. Overall, anti‐inflammatory drugs show promise in addiction treatment, although these strategies remain in the early stages of research.

### 4.3 Limitations

This study has several limitations. First, although WoSCC database only used by numerous bibliometric studies is the world's leading citation database and indexes high‐impact journals, ensuring article quality [59, 60], its selective coverage limits research comprehensiveness. Despite significant publication overlap across various databases, precise bibliometric analyses should incorporate additional sources such as PubMed. Second, only publications in English were included, which may introduce language‐based selection bias. Third, due to time constraints during the initial phase of literature selection, publications dated after January 1, 2025, were not considered in the findings. Fourth, inconsistencies may exist across various aspects. For example, an institution might use different names at different times. Fifth, citations are subject to multiple inherent biases, such as selective and arbitrary citation, mis‐citation, and honorary citation [61].

## 5 Conclusion

In summary, this study employed bibliometric methods to map the evolution of the research field focusing on SUDs and inflammation imbalance. The dominant keywords associated with SUDs and inflammation were “oxidative stress” and “neuroinflammation.” The most influential country in this field was the US. The most prolific author, institution, and journal were Maes, Michael, the University of California System, and *the JOURNAL OF ETHNOPHARMACOLOGY*, respectively. Notable future research directions include: (1) the role of neuroinflammation in the mechanisms and treatment of SUDs; (2) the impact of the gut‐immune‐brain axis on SUDs; (3) the potential application of anti‐inflammatory drugs like N‐acetylcysteine in drug addiction treatment. These findings offer a scientific foundation for understanding addiction mechanisms and therapeutic strategies.

## Author Contributions

Shirui Cao and Longtao Yang designed the study and wrote the manuscript. Ruixin Wang and Jun Liu provided critical revision of the manuscript for important intellectual content. All authors critically reviewed the content and approved the final version for publication.

## Funding

This work was supported by the Fundamental Research Funds for the Central Universities of Central South University 1053320230244; Open Project of Key Laboratory of Drug Monitoring and Control, Ministry of Public Security 2024‐KLDMC‐07; National Natural Science Foundation of China U22A20303; National Natural Science Foundation of China 82571705; Innovative Province Special Construction Foundation of Hunan Province 2019SK2131; the Science and Technology Innovation Program of Hunan Province 2021RC4016 and Clinical Research Center for Medical Imaging in Hunan Province in China 2020SK4001.

## Ethics Statement

The authors have nothing to report.

## Conflicts of Interest

The authors declare no conflicts of interest.

## Supporting information


**Figure S1:** Co‐authorship analysis of institutions (A) and authors (B) as well as reference co‐citation (C).

**Figure S2:** Keyword co‐occurrence networks centered on “substance use disorders” (A), “opioid” (B), “methamphetamine” (C), “cannabis” (D), and “cocaine” (E).

**Table S1:** Number of total citations in the top 10 literature over the past 10 years.

## Data Availability

The datasets used or analyzed during the current study are available from the corresponding author on reasonable request.
