# Supplementary figures and images for: Worldwide Research Trends on Substance Use Disorder‐Related Inflammatory Imbalances: A Bibliometric Analysis
Source: CNS Neurosci Ther. 2025 Dec 20;31(12):e70719. doi: 10.1002/cns.70719 (PMC12717472; doi:10.1002/cns.70719)

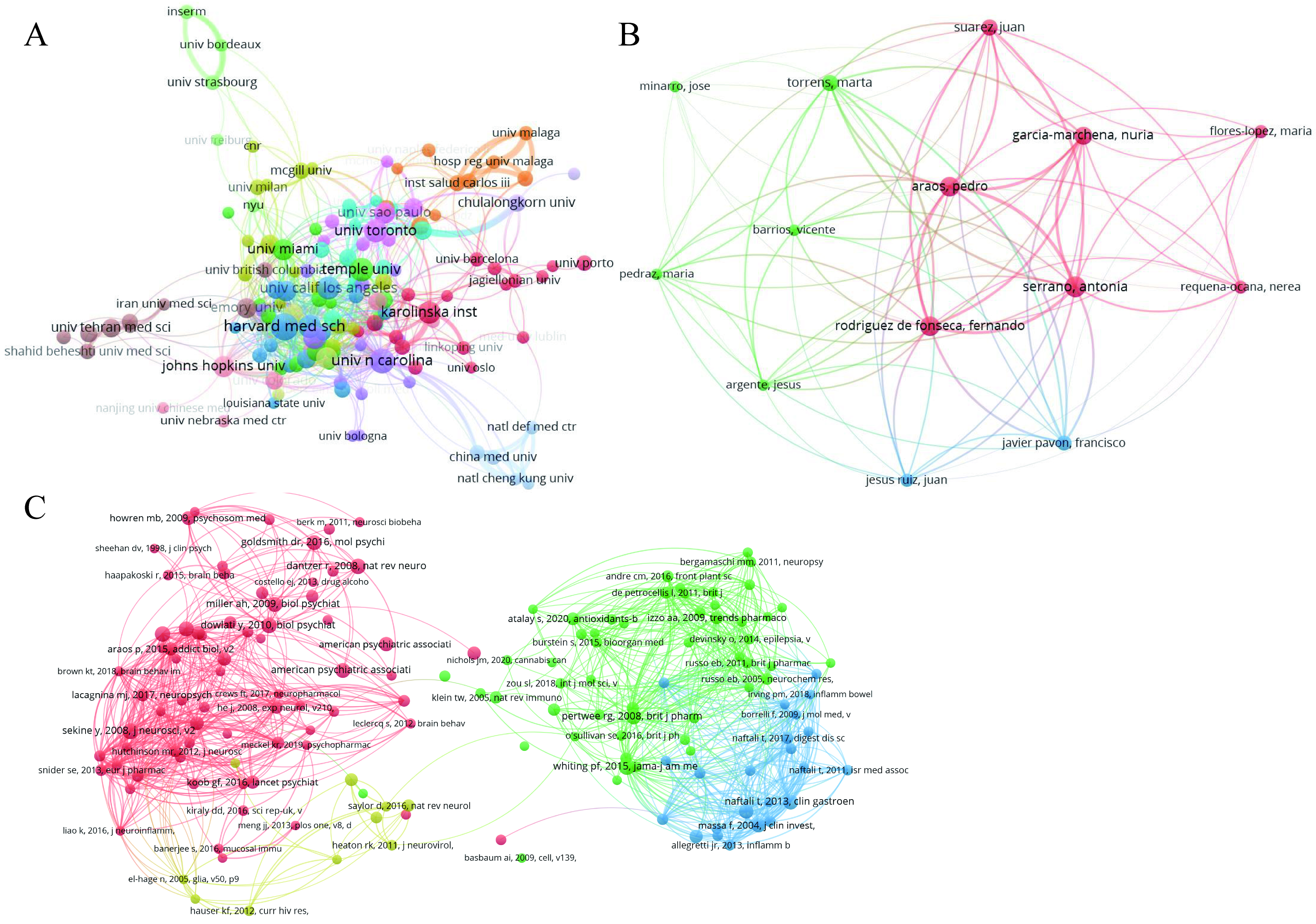

Supplement: Supplementary file 1 — Figure S1: Co‐authorship analysis of institutions (A) and authors (B) as well as reference co‐citation (C). [file CNS-31-e70719-s002.tif]

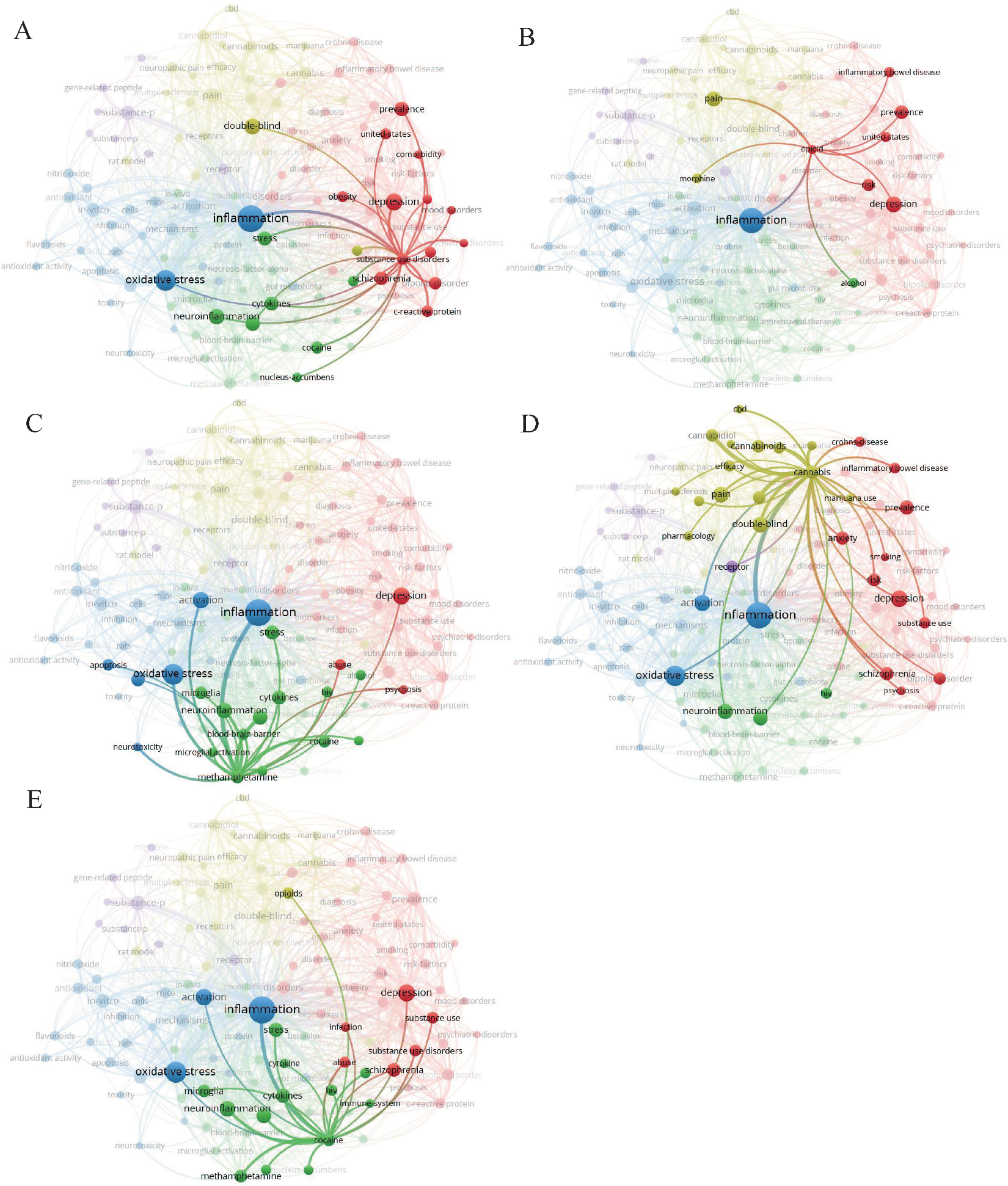

Supplement: Supplementary file 2 — Figure S2: Keyword co‐occurrence networks centered on “substance use disorders” (A), “opioid” (B), “methamphetamine” (C), “cannabis” (D), and “cocaine” (E). [file CNS-31-e70719-s001.tif]
